# The Hallmarks of Glioblastoma: Functional Interplay Between Long Non-Coding RNAs and RNA-Binding Proteins

**DOI:** 10.3390/cells15141251

**Published:** 2026-07-11

**Authors:** Karlijn A. van de Langerijt, Md Golam Kibria, Genaro R. Villa, Marco Mineo

**Affiliations:** 1 Harvey Cushing Neuro-Oncology Laboratories, Department of Neurosurgery, Brigham and Women’s Hospital, Boston, MA 02115, USA; 2 Harvard Medical School, Boston, MA 02115, USA

**Keywords:** Glioblastoma, long non-coding RNAs, RNA-binding proteins

## Abstract

Glioblastoma (GBM) is the most aggressive primary brain tumor, characterized by rapid progression, therapeutic resistance, and poor patient prognosis. Emerging evidence highlights the critical role of long non-coding RNAs (lncRNAs) in GBM pathogenesis, particularly through their interactions with RNA-binding proteins (RBPs). These interactions form complex regulatory networks that influence multiple GBM hallmarks, such as sustained proliferation, induction of angiogenesis, and immune evasion. Additionally, these interactions play a pivotal role in maintaining glioma stem-like cells, a subpopulation responsible for tumor recurrence and resistance to conventional therapies. Understanding the mechanistic basis of lncRNA-RBP interactions offers promising opportunities for therapeutic intervention. Targeting these networks could enable the development of novel and more effective treatment strategies. This review provides an in-depth analysis of the molecular mechanisms by which lncRNA-RBP complexes promote GBM development.

## 1. Introduction

Glioblastoma (GBM), the most aggressive primary malignant tumor of the adult central nervous system, is characterized by profound intratumoral heterogeneity, diffuse infiltration, and nearly universal therapeutic resistance [1,2,3]. Despite decades of study into genomics, epigenomics, and transcriptomics, clinical outcomes have improved only marginally, and median survival remains under two years. This highlights the urgent need to better understand the regulatory mechanisms that enable GBM cells to adapt, survive, and evolve under selective pressures imposed by the brain microenvironment and therapy [1,2,3].

A conceptual framework that has proven useful across oncology is the organization of tumor biology into functional “hallmarks,” such as sustained proliferative signaling, invasion of the surrounding tissue, metabolic reprogramming, and immune escape [4,5]. In GBM, these hallmark-like phenotypes are particularly pronounced and dynamically regulated, reflecting the tumor’s need to thrive in a metabolically constrained, immunologically specialized, and spatially heterogeneous environment [6]. While canonical genetic alterations in protein-coding genes contribute to these phenotypes, they do not fully account for the plasticity and resilience that define GBM biology.

In humans, a large fraction of the genome encodes thousands of long non-coding RNAs (lncRNAs). They have traditionally been defined as transcripts longer than 200 nucleotides (200 nt) that do not code for proteins, although the most recent consensus suggests defining lncRNAs as non-coding RNAs longer than 500 nt, mainly transcribed by RNA polymerase II (Pol II) [7]. Many of these lncRNAs undergo splicing and polyadenylation, and, with respect to protein-coding genes, they can be classified as intergenic, intronic, or antisense [8]. The GENECODE consortium estimates that the human genome contains more than 16,000 lncRNAs; however, other studies suggest the number exceeds 100,000 [9,10,11]. Notably, while the number of coding genes is similar across species with vastly different developmental and cognitive complexities, the number of lncRNAs increases alongside organismal complexity [12,13]. Complex organisms require more sophisticated regulatory mechanisms, in which lncRNAs act as key regulators of gene expression, especially during development and in the brain.

**Figure 1:**
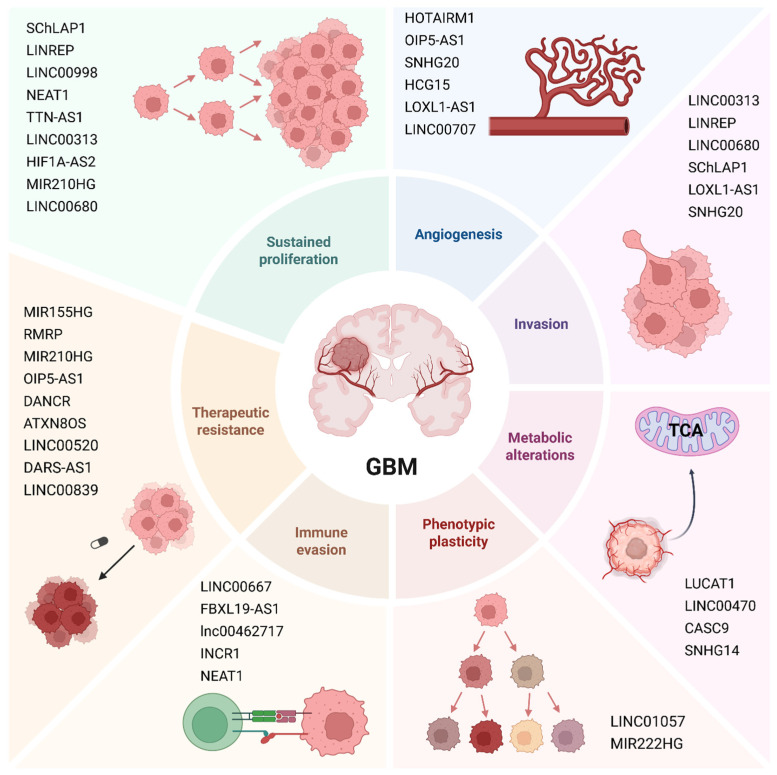
LncRNAs implicated in the hallmarks of GBM. The key hallmarks driving GBM progression (sustained proliferation, angiogenesis, invasion, metabolic alteration, phenotypic plasticity, therapeutic resistance, and immune evasion) are shown alongside the lncRNAs functionally associated with each hallmark. Created in BioRender. Mineo, M. (2026) https://BioRender.com/mb0zdal.

Mutation or aberrant expression of lncRNAs can cause the development and progression of cancer [14]. Several lncRNAs exhibit remarkable tumor and cell-lineage-specific expression and are increasingly recognized as modulators of oncogenic signaling, chromatin organization, and stress responses [15,16,17]. In GBM, numerous lncRNAs have been implicated in the maintenance of stem-like cellular states, invasion, angiogenesis, metabolic adaptation, immune modulation, and resistance to radiation and chemotherapy [18,19,20] (Figure 1). Importantly, many lncRNAs are dynamically regulated by hallmark-associated stresses such as hypoxia, inflammation, and genotoxic injury, positioning them as context-dependent sensors and amplifiers of tumor state rather than static biomarkers [21,22,23].

To fulfill their regulatory function, several lncRNAs interact with RNA-binding proteins (RBPs), assembling them into functional complexes, guiding them to specific transcripts, competing for binding, or modulating their localization and activity, thereby reshaping post-transcriptional regulatory networks in a context-dependent manner [24,25]. RBPs and lncRNAs govern multiple layers of RNA metabolism, including transcriptional output, alternative splicing, RNA stability, localization, and translation [17,26]. These ribonucleoprotein complexes enable rapid, context-dependent remodeling of gene expression programs, an attribute well-suited to the fluctuating microenvironmental conditions encountered by GBM cells. Unlike static genomic alterations, lncRNA-RBP networks are highly dynamic and reversible, enabling rapid transcriptional and post-transcriptional reprogramming in response to microenvironmental stress.

In this review, we examine the roles of lncRNA-RBP complexes through the lens of hallmark-like phenotypes in GBM (Table 1). By organizing emerging data around functionally defined tumor states, including hypoxic adaptation, stemness, metabolic rewiring, immune evasion, invasion, and therapeutic resistance, we aim to highlight unifying principles by which post-transcriptional regulation supports GBM malignancy. We further discuss how these regulatory axes may expose vulnerabilities that may not be evident from DNA-centric analyses alone, offering new opportunities for therapeutic intervention in a disease that remains incurable.

## 2. Sustained Proliferation

Proliferation is a central hallmark of GBM, enabling the rapid and sustained expansion of the tumor and supporting disease progression [57]. GBM displays exceptionally high proliferative capacity, supported by widespread dysregulation of growth-promoting pathways. Despite advances in characterizing these pathways, their intertwined nature has limited clinical efficacy and underscores the need for deeper mechanistic insight [58,59]. GBM proliferation, like that of many tumors, is often described as “uncontrolled” due to the disruption of key cell cycle regulatory genes. These alterations impair cancer cells’ ability to exit the cell cycle properly, resulting in sustained, continuous division. However, cell cycle progression remains a tightly coordinated process, and certain checkpoints must retain functionality to support ongoing proliferation [60]. In this context, lncRNAs and RBPs have emerged as critical regulators of cell cycle dynamics, helping maintain sufficient control to prevent mitotic catastrophe while still enabling rapid tumor growth. The interplay between lncRNAs and RBPs constitutes a central layer of post-transcriptional regulation, governing transcript abundance and stability in ways that reinforce oncogenic proliferative programs.

**Figure 2:**
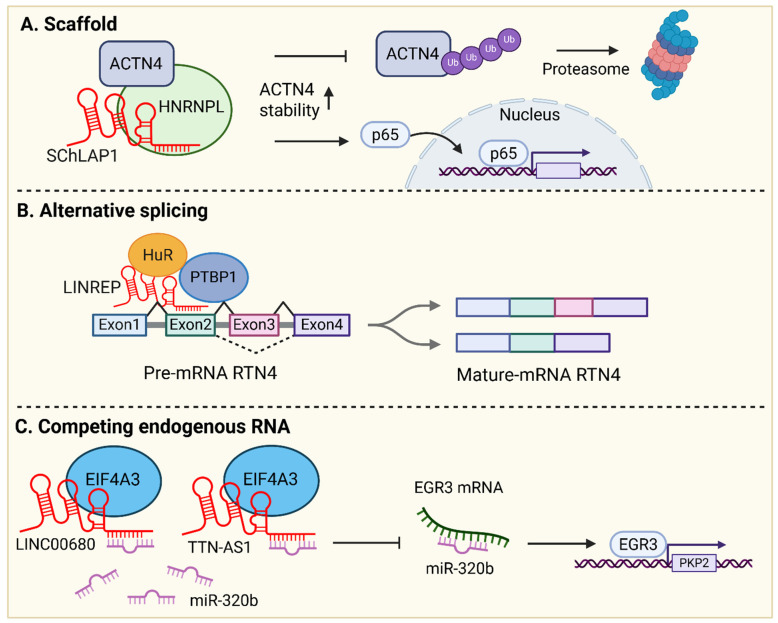
Mechanisms by which lncRNA-RBP interactions drive sustained proliferation in GBM. (**A**) SChLAP1 scaffolds HNRNPL and ACTN4, stabilizing ACTN4 against proteasomal degradation. This stabilization promotes nuclear accumulation of the NF-κB subunit p65, thereby activating NF-κB signaling. (**B**) LINREP promotes alternative splicing by increasing PTBP1 binding to RTN4 transcripts, leading to skipping of exon 3. (**C**) LINC00680 and TTN-AS1 act as competing endogenous RNAs by sequestering miR-320b, thereby relieving repression of EGR3 mRNA. Elevated EGR3, in turn, drives upregulation of PKP2 expression. Created in BioRender. Mineo, M. (2026) https://BioRender.com/b0n2app.

A subset of oncogenic lncRNAs in GBM exerts its proliferative effects by stabilizing RBPs. One example is the SWI/SNF complex antagonist associated with prostate cancer 1 (SChLAP1), which is overexpressed in GBM. SChLAP1 depletion significantly inhibited tumor cell growth in vitro and in vivo, leading to a survival benefit. Mechanistically, SChLAP1 functions by scaffolding the RBP heterogeneous nuclear ribonucleoprotein L (HNRNPL) and actinin alpha 4 (ACTN4), thereby stabilizing ACTN4 and preventing its proteasomal degradation (Figure 2A). Stabilized ACTN4 promotes nuclear accumulation of the NF-κB subunit p65 and subsequent activation of NF-κB signaling, a pathway strongly associated with sustained proliferation [27]. The RBP polypyrimidine tract-binding protein 1 (PTBP1), a central regulator of oncogenic splicing programs, is also regulated through lncRNA-dependent mechanisms. The lncRNA LINREP is upregulated in GBM and binds PTBP1. By promoting the formation of the PTBP1/human antigen R (HuR, ELAVL1) complex, LINREP protects PTBP1 from ubiquitin-proteasome degradation. LINREP also promotes the dissociation of nuclear UPF1 RNA helicase and ATPase (UPF1) from PTBP1, thereby increasing PTBP1 binding to reticulon 4 (RTN4) transcripts and enhancing RTN4 exon 3 skipping (Figure 2B). This dual mechanism of stabilizing PTBP1 while amplifying its splicing function drives oncogenic alternative splicing and promotes aggressive tumor progression [28]. Importantly, not all lncRNA-RBP interactions promote malignancy, as some lncRNAs serve as tumor suppressors by opposing proliferative signals. LINC00998 directly binds Chromobox 3 (CBX3), preventing its ubiquitin-mediated degradation and thereby increasing CBX3 protein levels. CBX3 is a multifunctional protein that acts as an RNA-binding protein in addition to its roles in chromatin regulation. The increased levels of CBX3 enhance H3K9me3-dependent repression at oncogenic promoters such as c-Met. This repression inhibits Akt/mTOR signaling and limits GBM cell proliferation [29].

These studies underscore a recurring paradigm in which lncRNAs act as stabilizing scaffolds, protecting RBPs from proteasomal degradation. By preserving the activity of factors such as ACTN4, PTBP1, or UPF1, these lncRNAs sustain oncogenic signaling pathways that promote GBM proliferation. In contrast, several GBM-associated lncRNAs depend on specific RBPs to regulate their own stability. The RBP serine and arginine-rich splicing factor 1 (SRSF1) supports proliferation by stabilizing nuclear paraspeckle assembly transcript 1 (NEAT1), a lncRNA that promotes cell-cycle progression through upregulation of cyclins and cyclin-dependent kinases (CDKs). Silencing SRSF1 reduces NEAT1 levels, leading to G1 arrest and inhibition of proliferation, confirming NEAT1 as a major downstream effector [30]. The RBP eukaryotic translation initiation factor 4A3 (EIF4A3) stabilizes the oncogenic lncRNAs LINC00680 and TTN antisense RNA 1 (TTN-AS1), which are significantly overexpressed in GBM tissues and cell lines. Functional studies showed that knocking down EIF4A3, LINC00680, or TTN-AS1 individually significantly reduced proliferation. Importantly, co-silencing of EIF4A3 with either LINC00680 or TTN-AS1 produced a greater inhibition of proliferation than single knockdowns, suggesting synergistic therapeutic potential. Mechanistically, LINC00680 and TTN-AS1 act as competing endogenous RNAs (ceRNAs) that bind to miR-320b, preventing its interaction with early growth response 3 (EGR3) mRNA (Figure 2C). The resulting accumulation of EGR3 upregulates plakophilin 2 (PKP2) expression, a known activator of the EGFR signaling pathway, which is a key driver of proliferation in GBM [31,61]. UPF1 is also a critical binding partner of LINC00313, and both molecules are upregulated in GBM tissues and cell lines. UPF1 binds directly to LINC00313 and stabilizes it, thereby enhancing its abundance. The stabilized LINC00313 functions as a ceRNA, sequestering the tumor suppressor microRNAs (miRNAs) miR-342-3p and miR-485-5p, thereby relieving repression of the transcription factor Zic family zinc finger 4 (Zic4). In turn, Zic4 binds to the promoters of both UPF1 and Linc-00313, thereby upregulating their expression and establishing a positive feedback loop. Zic4 also binds the promoter of SHC binding and spindle-associated 1 (SHCBP1) to promote its transcription. SHCBP1 contributes to malignant phenotypes by activating the MEK/ERK signaling pathway [32]. These findings identify RBPs as the primary regulators of proliferative signaling, suggesting that targeting RBP-lncRNA stabilization could break the feedback loops that maintain GBM proliferation.

A defining characteristic of GBM is its ability to adapt and sustain proliferation under hypoxic stress [62,63]. Cellular adaptation to low oxygen levels in GBM involves widespread changes in gene expression, with interactions between lncRNAs and RBPs playing a central regulatory role. A notable example of a hypoxia-driven lncRNA-RBP axis is hypoxia-inducible factor 1 alpha-antisense RNA 2 (HIF1A-AS2), which is selectively upregulated in mesenchymal-like GBM cells. HIF1A-AS2 supports stem-like properties and cell proliferation by scaffolding the RBPs Insulin-like Growth Factor 2 mRNA-Binding Protein 2 (IGF2BP2) and ATP-dependent RNA helicase A (DHX9), thereby stabilizing and increasing the expression of oncogenic targets such as high mobility group AT-hook 1 (HMGA1) [21]. This interaction enables cells to adapt effectively to hypoxic environments, reinforcing their malignant traits. Another prominent hypoxia-responsive lncRNA is MIR210 host gene (MIR210HG), a critical mediator of hypoxia-driven GBM aggressiveness. Its expression strongly correlates with hypoxia signatures and poor patient survival. Under low-oxygen conditions, MIR210HG enhances the activity of the transcription factor POU class 2 homeobox 1 (POU2F1/OCT1), which in turn upregulates oncogenic targets such as Insulin-like Growth Factor Binding Protein 2 (IGFBP2) [64].

Overall, lncRNA-RBP interactions emerge as critical regulators of GBM proliferation by shaping gene expression at multiple levels, including RNA metabolism and protein turnover. Through these interactions, lncRNAs can function either as oncogenic drivers, stabilizing pro-proliferative factors and amplifying signaling pathways such as EGFR, or as tumor suppressors that restrain aberrant growth by dampening these same pathways. Collectively, these mechanisms underscore the versatility and centrality of lncRNA-RBP interactions in GBM and highlight multiple avenues of therapeutic vulnerability.

## 3. Angiogenesis

A key feature that protects the brain is the blood–brain barrier (BBB). The BBB is a highly specialized neurovascular structure composed of brain endothelial cells, astrocytes, pericytes, and junctional complexes, including tight and adherens junctions [65]. While blocking harmful substances and maintaining a stable environment for proper brain function, the BBB also makes it more challenging to deliver certain medications to the brain. In GBM, this barrier is disrupted, resulting in a heterogeneous blood-tumor barrier (BTB) with non-uniform permeability [66]. This disruption is driven in part by angiogenesis, a crucial hallmark of GBM pathophysiology, which enables the sustained tumor growth necessary for disease progression [57]. GBMs are among the most highly vascularized solid tumors, characterized by abnormal vascular features that distinguish them from normal brain vasculature. The abnormal vasculature with tortuous, dilated, and chaotically organized architecture results in inefficient blood flow and hypoxic microenvironments [67]. While pro-angiogenic drivers have been well established, therapeutic targeting of these pathways has yielded limited clinical benefit. Notably, the AVAglio and RTOG 0825 trials demonstrated that bevacizumab, a monoclonal antibody against vascular endothelial growth factor A, improved progression-free survival (PFS) but failed to improve overall survival (OS) [68,69]. A meta-analysis including over 3300 patients confirmed this pattern: anti-angiogenic therapy prolongs PFS but confers little OS benefit [70]. This suggests that additional regulatory mechanisms are at play. Growing evidence indicates that lncRNAs and RBPs form intricate post-transcriptional networks that control angiogenic and vasculogenic mimicry (VM), contributing to the vascular plasticity and characteristics of GBM.

**Figure 3:**
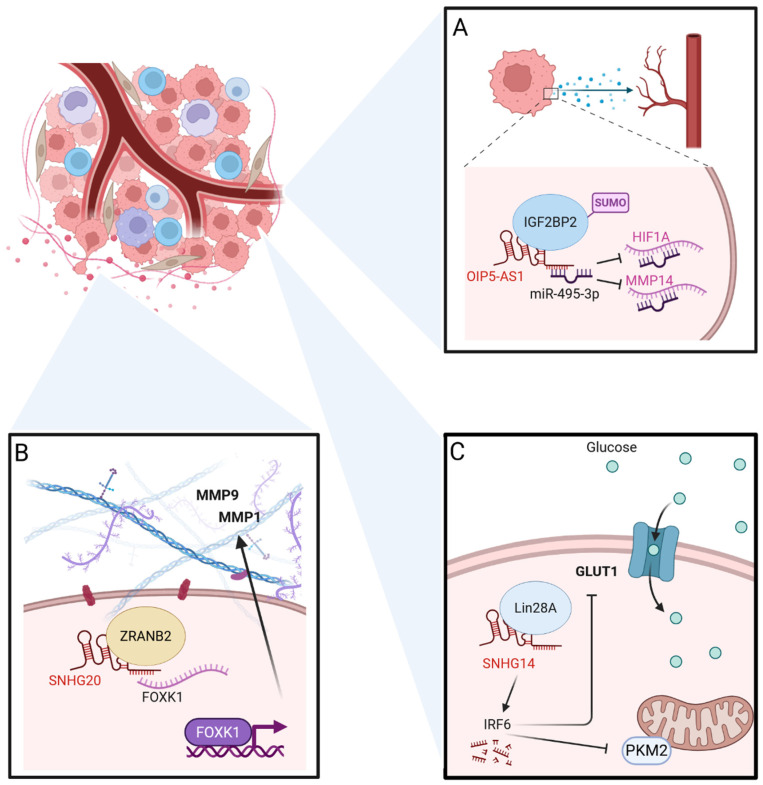
Examples of mechanisms by which lncRNA-RBP interactions promote angiogenesis, invasion, and metabolic alterations in GBM. (**A**) SUMOylated IGF2BP2 stabilizes OIP5-AS1, and the resulting complex sequesters miR-495-3p, thereby relieving repression of the angiogenic effectors HIF1A and MMP14. (**B**) ZRANB2 stabilizes SNHG20, which in turn prevents degradation of FOXK1 mRNA, thereby promoting transcriptional activation of MMP1 and MMP9. (**C**) Lin28A stabilizes SNHG14, thereby facilitating the degradation of IRF6 mRNA. Loss of IRF6 relieves transcriptional repression of GLUT1 and PKM2. Created in BioRender. Mineo, M. (2026) https://BioRender.com/k9cek48.

A central example of such regulatory complexity is the METTL3/HOTAIRM1/IGFBP2 axis. The Methyltransferase-Like Protein 3(METTL3) promotes the stabilization of the lncRNA HOXA Transcript Antisense RNA, Myeloid-Specific 1 (HOTAIRM1), which in turn binds the mRNA of IGFBP2, enhancing its expression. All three components are significantly upregulated in GBM and collectively promote VM formation [33]. This pathway illustrates how an m6A-dependent lncRNA-RBP feedback loop can enhance pro-angiogenic signaling and contribute to therapy resistance. An RBP with a similar name but fundamentally different in structure, location, and function, IGF2BP2, drives VM through lncRNA-miRNA interaction. Small Ubiquitin-like Modifier (SUMO) ylation increases IGF2BP2 stability, allowing it to bind and stabilize the oncogenic lncRNA OIP5 Antisense RNA 1 (OIP5-AS1) more effectively. The SUMO-IGF2BP2/OIP5-AS1 complex acts as a sponge for miR-495-3p, releasing downstream angiogenic effectors and promoting VM (Figure 3A). Both IGF2BP2 and OIP5-AS1 are significantly upregulated in GBM [34]. Together with METTL3, IGF2BP2 exemplifies how RBPs integrate diverse lncRNA signals to remodel the glioma vasculature.

Zinc finger RANBP2-type containing 2 (ZRANB2) is another oncogenic RBP that stabilizes the lncRNA Small Nucleolar RNA Host Gene 20 (SNHG20), allowing it to sequester suppressive miRNAs and increase Forkhead Box K1 (FOXK1) expression. This cascade promotes VM formation and is significantly upregulated in GBM [36]. Similarly, elevated expression of poly (A)-binding protein cytoplasmic 5 (PABPC5) in GBM stabilizes human leukocyte antigen complex group 15 (HCG15), which recruits Staufen double-stranded RNA-binding protein 1 (STAU1) to accelerate the degradation of the tumor suppressor zinc-finger protein 331 (ZNF331). The resulting increase in extracellular matrix (ECM) remodeling and VM-related gene expression is reduced when PABPC5, HCG15, or STAU1 is inhibited [37]. In contrast, TIA1-related protein (TIAR) functions as a tumor-suppressive RBP. Under normal conditions, TIAR destabilizes the oncogenic lncRNA LOXL1 antisense RNA 1 (LOXL1-AS1), thereby limiting its accumulation. However, TIAR downregulation permits persistent LOXL1-AS1 expression, which suppresses miR-374b-5p and consequently increases matrix metallopeptidase 14 (MMP14) levels, promoting VM [38]. The reciprocal pattern of LOXL1-AS1 upregulation and TIAR loss suggests that VM is driven not only by oncogenic gain-of-function pathways but also by the disruption of suppressive RBP-mediated regulatory mechanisms. Additional RBPs further support VM by coordinating multiple post-transcriptional networks. For example, heterogeneous nuclear ribonucleoprotein D (HNRNPD), in cooperation with the transcription factor Zinc Fingers and Homeoboxes 2 (ZHX2), upregulates long intergenic non-coding RNA 707 (LINC00707). LINC00707 functions as a ceRNA that sequesters miR-651-3p, relieving repression of the transcription factor SP2, a regulator implicated in ECM remodeling and VM formation. Elevated HNRNPD and LINC00707 expression in GBM is associated with enhanced tubular network formation, highlighting the contribution of an HNRNPD/LINC00707/miR-651-3p/SP2 regulatory axis to vascular plasticity [39].

These findings collectively reveal that interactions between lncRNAs and RBPs form an interconnected network that governs angiogenesis and VM in GBM. Various upstream signals, such as m6A modifications, post-translational modifications of RBPs, and the loss of tumor-suppressive RBP functions, converge on a common downstream outcome: enhanced ECM remodeling, increased vascular plasticity, and the formation of a therapy-resistant VM niche. This recurrent convergence highlights that VM is driven not by isolated factors but by a coordinated, multilayered post-transcriptional network. Notably, the upregulation of oncogenic lncRNAs and RBPs, alongside the loss of suppressive RBPs, underscores their significance as biomarkers of GBM aggressiveness. Furthermore, the lncRNA-RBP networks present promising new therapeutic targets, especially where traditional anti-angiogenic therapies are ineffective.

## 4. Invasion

Invasion, a canonical hallmark of cancer, is the ability of tumor cells to escape the primary tumor mass and invade nearby tissues through active migration and ECM degradation [57]. GBM tumor cells display an aggressive, invasive phenotype that allows them to infiltrate the surrounding brain parenchyma along white matter tracts and perivascular spaces [71,72]. This pattern of invasion sets GBM apart from lower-grade gliomas and represents a major barrier to curative treatment, as infiltrating cells escape surgical resection and lead to recurrence [71,73]. The invasive phenotype arises from an intricate molecular network that coordinates cytoskeletal reorganization, ECM remodeling, and dynamic crosstalk between tumor cells and the tumor microenvironment (TME) [74,75]. Emerging evidence suggests that lncRNAs and RBPs are crucial in coordinating these processes by regulating key signaling pathways and effector proteins that govern GBM cell motility and tissue infiltration.

Several lncRNA-RBP interactions initially characterized in the context of proliferation also contribute to the invasive phenotype of GBM. This functional overlap reflects the convergence of proliferative and invasive programs on shared downstream pathways, including cytoskeletal remodeling, activation of pro-survival signaling cascades, and suppression of cell–cell adhesion programs [75]. The UPF1/LINC00313, EIF4A3/LINC00680, and SChLAP1/HNRNPL/ACTN4 axes are notable examples of this duality, whereby molecular networks that enhance proliferative capacity simultaneously promote GBM cell migration and invasion. These observations highlight the ability of lncRNA-RBP regulatory circuits to coordinate multiple malignant phenotypes.

Consistent with this concept, EIF4A3-mediated stabilization of LINC00680 and TTN-AS1 enhances the invasive capacity of GBM cells. Functional studies have demonstrated that silencing EIF4A3, LINC00680, or TTN-AS1 individually significantly reduces invasion, whereas combined knockdown of EIF4A3 with either lncRNA produces an even greater inhibitory effect [31]. Similarly, depletion of either UPF1 or LINC00313 significantly impairs GBM cell migration and invasion, underscoring the importance of this RBP-lncRNA network in maintaining invasive behavior. In this pathway, the downstream effector SHCBP1, activated through a Zic4-mediated feedback loop, further promotes cellular motility [32]. The SChLAP1/HNRNPL/ACTN4 axis likewise appears to extend beyond proliferation to influence GBM invasion, potentially through the NF-κB signaling pathway [27]. However, while this axis has been implicated as a driver of invasion, direct experimental validation of its effects on migration and invasion remains lacking. Further studies are needed to clarify its functional contribution to GBM invasiveness.

LncRNA-RBP networks involved in GBM angiogenesis also contribute to invasion, underscoring the close mechanistic relationship between these hallmarks of malignancy. Both processes rely extensively on ECM remodeling, while hypoxia arising from aberrant tumor vasculature serves as a potent inducer of invasive behavior [76]. By simultaneously regulating neovascularization and invasive behavior, these networks facilitate GBM progression. One such network involves ZRANB2-mediated stabilization of SNHG20, which prevents degradation of FOXK1 mRNA (Figure 3B). Knockdown of ZRANB2 significantly inhibits GBM cell migration and invasion, highlighting its essential role in promoting invasive behavior. The downstream effector FOXK1 further enhances invasion by transcriptionally activating MMP1 and MMP9, key matrix metalloproteinases involved in extracellular matrix degradation and glioma cell dissemination [36]. In contrast, TIAR suppression of the lncRNA LOXL1-AS1 significantly reduces GBM cell migration and invasion [38].

Together, these findings demonstrate that lncRNA-RBP interactions serve as regulatory axes that collectively drive proliferative, angiogenic, and invasive programs in GBM through multiple downstream mechanisms. This dual role emphasizes the close connection between these cancer hallmarks.

## 5. Metabolic Alteration

GBM exhibits profound metabolic reprogramming that provides the nutrients and energy required to sustain rapid tumor growth and survival within a hostile microenvironment [77]. One of the most extensively studied metabolic alterations in GBM is the Warburg effect, in which tumor cells preferentially utilize aerobic glycolysis, leading to increased glucose uptake and lactate production despite oxygen availability [78]. In addition, GBM cells rely on glutaminolysis to replenish tricarboxylic acid (TCA) cycle intermediates and support biosynthetic pathways [79]. Altered lipid metabolism is another hallmark of GBM, facilitating membrane biosynthesis, energy storage, and cellular signaling processes that promote tumor progression [80,81,82]. Furthermore, mitochondrial function in GBM is reprogrammed toward anabolic metabolism rather than efficient ATP production [83]. These metabolic adaptations are frequently driven by oncogenic mutations and are further shaped by hypoxic conditions within the tumor microenvironment, collectively promoting tumor growth, cellular plasticity, and therapeutic resistance. Beyond oncogenic alterations, accumulating evidence indicates that the interplay between lncRNAs and RBPs plays a crucial role in regulating metabolic reprogramming in GBM [84].

Hypoxia is a defining feature of GBM and profoundly influences tumor metabolism, including the enhancement of aerobic glycolysis, by regulating multiple transcriptional programs. A recent study identified lung cancer-associated transcript 1 (LUCAT1) as one of the most highly expressed lncRNAs in glioma stem cells (GSCs) under hypoxic conditions. LUCAT1 expression positively correlates with hypoxia-inducible factor-1α (HIF-1α) levels and is required for the expression of HIF-1α target genes [40]. Mechanistically, LUCAT1 directly interacts with HIF-1α and facilitates its association with the RBP coactivator CBP/p300, thereby enhancing hypoxia-responsive gene transcription. Consistent with this mechanism, LUCAT1 knockdown markedly impairs formation of the HIF-1α–CBP/p300 complex and attenuates hypoxia-induced transcriptional activity [40].

Because of their elevated metabolic demands, tumor cells require increased glucose uptake to sustain continuous energy production and biosynthesis. Glucose uptake, the first step in cellular energy metabolism, is mediated by glucose transporters (GLUTs) [85]. The Lin28A/SNHG14/IRF6 regulatory axis plays a critical role in metabolic reprogramming and glioma progression by modulating the expression of GLUT1 and pyruvate kinase M2 (PKM2) [41]. Both the RBP lin-28 RNA-binding posttranscriptional regulator A (Lin28A) and the lncRNA small nucleolar RNA host gene 14 (SNHG14) are highly expressed in glioma, whereas interferon regulatory factor 6 (IRF6) is downregulated. Mechanistically, Lin28A enhances SNHG14 stability and expression, and elevated SNHG14 promotes STAU1-mediated degradation of IRF6 mRNA by interacting with its 3′ untranslated region. Reduced IRF6 expression subsequently relieves transcriptional repression of GLUT1, a major glucose transporter, and PKM2, a key enzyme that promotes aerobic glycolysis (Figure 3C). Consequently, this axis enhances both glucose uptake and glycolytic flux. PKM2 expression is also regulated by the RBP PTBP1. Elevated PTBP1 expression in GBM, together with heterogeneous nuclear ribonucleoprotein A1 (HNRNPA1) and HNRNPA2, increases the PKM2/PKM1 ratio, thereby favoring aerobic glycolysis and supporting tumor metabolism [86].

Beyond glucose uptake, the activity and stability of glycolytic enzymes critically determine the overall rate of glycolysis. Several glycolytic enzymes act as rate-limiting factors regulating metabolic flux through the pathway. Hexokinase (HK), which catalyzes the phosphorylation of glucose to glucose-6-phosphate, represents the first rate-limiting step of glycolysis and exists in four isoforms (HK1–HK4) [87,88]. Among these, HK2 plays a predominant role in promoting aerobic glycolysis in cancer cells [89]. Growing evidence indicates that lncRNA-RBP complexes substantially contribute to the regulation of glycolytic enzyme expression and stability in GBM. One well-characterized example is the interaction between IGF2BP2 and the lncRNA cancer susceptibility candidate 9 (CASC9). CASC9 is significantly upregulated in GBM tissues and contains an m6A-modified RRACH motif. This m6A modification is recognized by the m6A reader IGF2BP2, thereby enhancing CASC9 stability. The resulting IGF2BP2/CASC9 complex subsequently binds to the m6A-modified site within the 3′ untranslated region of HK2 mRNA and markedly increases its stability, whereas IGF2BP2 alone exerts only a modest stabilizing effect [42].

In addition to regulating glycolytic enzyme transcripts, lncRNA-RBP interactions also influence protein stability, which is critical for maintaining glycolytic activity. Since the ubiquitin–proteasome system is responsible for the degradation of the majority of intracellular proteins, its regulation can profoundly affect metabolic pathways. A representative example is lncRNA LINC00470, which is overexpressed in GBM and promotes AKT phosphorylation. Notably, LINC00470 does not directly interact with AKT. Instead, the RBP FUS simultaneously binds LINC00470 and AKT, forming a ternary complex that retains FUS in the cytoplasm and enhances AKT activation. Increased AKT phosphorylation subsequently protects HK1 from ubiquitin-mediated degradation, thereby sustaining glycolytic activity and promoting metabolic adaptation in GBM cells [43].

## 6. Phenotypic Plasticity

GBM cellular plasticity refers to the ability of tumor cells to undergo phenotypic and molecular changes during progression, driven by complex tumor microenvironmental factors, epigenetic modifications, and selective pressures from radiotherapy or chemotherapy [90]. This plasticity underlies profound intratumoral heterogeneity and therapeutic resistance. Early efforts to capture this heterogeneity led to the classification of GBM into four molecular subtypes: proneuronal, neural, classical, and mesenchymal [2]. Transcriptomic analyses of patient tumors and patient-derived GBM cells further revealed that lncRNA expression is subtype-specific, highlighting the role of lncRNAs in shaping cellular phenotypes [15,91,92]. More recently, single-cell transcriptomic studies have refined this view by showing that individual GBMs contain malignant cells occupying a limited set of recurrent cellular states, including neural-progenitor-like (NPC-like), oligodendrocyte-progenitor-like (OPC-like), astrocyte-like (AC-like), and mesenchymal-like (MES-like) programs [93]. Building on this, spatial transcriptomics has demonstrated that these states are not randomly distributed but are organized into distinct local microenvironments, each typically enriched for a dominant cellular state. Notably, specific pairs of cellular states preferentially co-localize across multiple spatial scales, and these interactions are conserved across tumors [94]. Together, these pairwise relationships give rise to a higher-order tumor architecture composed of spatial layers, with hypoxia emerging as a key organizing principle. Importantly, cell states are not fixed, and interactions between lncRNAs and RBPs form regulatory networks that influence RNA stability, alternative splicing, subcellular localization, and epigenetic modifications, collectively enabling dynamic transitions between cellular states and contributing to intratumoral heterogeneity.

A key manifestation of GBM plasticity is the proneural-to-mesenchymal transition (PMT), a process associated with increased invasiveness, immunosuppression, and treatment resistance [95,96]. LINC01057 is overexpressed in GBM, particularly in the mesenchymal subtype, where it promotes mesenchymal differentiation by activating NF-κB signaling [44]. Predominantly localized in the nucleus, LINC01057 facilitates the translocation of IKKα into the nucleus, where IKKα phosphorylates the p65 subunit, enhancing its DNA-binding activity and transcriptional output. In addition, nuclear IKKα directly phosphorylates histone H3 at serine 10, a modification that promotes subsequent acetylation at lysine 14 by the associated histone acetyltransferase CREB-binding protein (CBP). These coordinated chromatin modifications increase accessibility at NF-κB-responsive promoters, thereby amplifying gene expression. Functionally, silencing LINC01057 in mesenchymal-like cells reduces expression of mesenchymal gene signatures while inducing proneural programs, whereas its overexpression in proneural-like cells drives the opposite shift. Notably, knockdown of IKKα abrogates the effects of LINC01057 overexpression on PMT, underscoring IKKα’s critical role in this regulatory axis [44].

Beyond direct NF-κB modulation, lncRNA-RBP interactions drive MES-like transition through epigenetic reprogramming mechanisms. A representative example is the MIR222HG/YWHAE/HDAC5 regulatory axis [45]. The lncRNA miR222/221 cluster host gene (MIR222HG), transcriptionally induced by the Spi-1 proto-oncogene (SPI1), acts as a molecular scaffold that bridges the non-canonical RBP tyrosine 3-monooxygenase/tryptophan 5-monooxygenase activation protein epsilon (YWHAE/14-3-3ε) with the histone deacetylase HDAC5. Through this interaction, MIR222HG facilitates the recruitment and stabilization of the YWHAE/HDAC5 complex at specific chromatin loci, leading to histone H4 deacetylation and consequent chromatin compaction. This epigenetic remodeling suppresses proneural gene expression programs while promoting mesenchymal transcriptional signatures, thereby driving PMT in GSCs [45]. Functionally, disruption of the MIR222HG axis impairs HDAC5-mediated repression, restores a more differentiated/proneural state, and attenuates mesenchymal features, highlighting how lncRNA-guided RBP complexes can orchestrate stable yet reversible cell state transitions through chromatin-level regulation.

## 7. Therapeutic Resistance

Therapeutic resistance remains one of the biggest challenges in GBM treatment, with the majority of patients relapsing despite the standard of care consisting of maximal surgical removal followed by radiation, chemotherapy, and Tumor-Treating Fields (TTFs) [97,98]. There are multiple mechanisms underlying this resistance, including both intrinsic and extrinsic tumor cell properties. Intrinsic tumor cell properties include enhanced DNA damage repair, dysregulated apoptosis, and metabolic reprogramming. Extrinsic factors include the immunosuppressive TME and the BBB, which limit drug delivery and bioavailability [98,99,100]. In addition, hypoxia plays a critical role in maintaining GSCs, which are central to therapy resistance and tumor recurrence [101]. Emerging evidence highlights the contribution of lncRNAs and their interactions with RBPs to the promotion of both chemo- and radio-resistance in GBM, underscoring their importance as regulators of GBM therapy response.

### 7.1. Chemoresistance

The Wnt/β-catenin pathway is frequently implicated in lncRNA-RBP-mediated temozolomide (TMZ) resistance, with multiple independent regulatory networks converging on its activation. One such lncRNA is the MIR155 host gene (MIR155HG), whose high expression correlates with poor patient survival in GBM. Both in vivo and in vitro studies showed that MIR155HG overexpression promotes TMZ resistance, whereas its knockdown enhances drug sensitivity. Mechanistically, MIR155HG functions as a scaffold by binding PTBP1, thereby regulating the expression of key components of the Wnt/β-catenin pathway [46,102,103]. Another lncRNA, the RNA component of the mitochondrial RNA processing endoribonuclease (RMRP), also contributes to Wnt/β-catenin pathway activation. RMRP is upregulated in both primary and recurrent GBM and interacts with the RBP insulin-like growth factor 2 mRNA-binding protein 3 (IGF2BP3) to reduce the stability of zinc and ring finger 3 (ZNRF3) mRNA, a negative regulator of Wnt/β-catenin signaling. Functional studies show that RMRP silencing reduces TMZ resistance and suppresses GBM growth in vitro and in vivo, highlighting its potential clinical relevance [47].

Hypoxia-induced lncRNAs play a critical role in multiple aspects of GBM progression, including cell proliferation, invasion, and resistance to therapy. As discussed above, MIR210HG, by interacting with the transcription factor OCT1, leads to upregulation of downstream targets, including IGFBP2 and fibroblast growth factor receptor 1 (FGFR1), both of which are key drivers of GBM sustained proliferation [64]. Elevated IGFBP2 expression contributes to therapy resistance by activating integrin β1-dependent ERK signaling, a pathway known to support cell survival, proliferation, and TMZ resistance. Similarly, FGFR1 signaling further reinforces proliferative and pro-survival pathways, thereby collectively enhancing the treatment resistance of GBM cells [64]. The RBP IGF2BP2 emerges as a key regulator in two mechanistically distinct lncRNA-mediated resistance pathways in GBM. In the first, IGF2BP2 directly binds and stabilizes the lncRNA OIP5-AS1. In TMZ-treated GBM cells, OIP5-AS1 is upregulated and functions as a competitive endogenous RNA that suppresses miR-129-5p. Functionally, knockdown of OIP5-AS1 increased sensitivity to TMZ in both in vitro and in vivo models, whereas its overexpression promoted resistance in vitro [35]. In a second, mechanistically distinct pathway, IGF2BP2 acts as an m6A reader, binding and stabilizing differentiation-antagonizing non-protein-coding RNA (DANCR), thereby increasing its abundance. In turn, DANCR interacts with forkhead box O1 (FOXO1) to promote its ubiquitination and degradation. Loss of FOXO1 prevents the transcriptional activation of phosphotyrosine interaction domain containing 1 (PID1), a tumor suppressor that enhances chemotherapy-induced apoptosis in GBM cells via NF-κB signaling [48,104]. Consistent with this model, silencing IGF2BP2 reduced cell viability in both etoposide-sensitive and etoposide-resistant cell lines [48]. Together, these findings illustrate how oncogenic RBPs can promote therapeutic resistance by stabilizing lncRNAs. However, the clinical relevance of the DANCR axis is somewhat limited by reliance on etoposide-based models, as etoposide is not part of standard GBM treatment regimens, which primarily rely on TMZ [58,97]. Future studies using TMZ-based resistance models will be essential to establish the translational significance of this pathway.

An emerging mechanism of chemoresistance involves the suppression of ferroptosis, a form of regulated cell death driven by iron-dependent lipid peroxidation. The lncRNA ataxin-8 opposite strand (ATXN8OS) is significantly downregulated in TMZ-resistant GBM cells, and its loss permits increased activity of the RNA-editing enzyme adenosine deaminase acting on RNA (ADAR). Elevated ADAR activity alters glutaminase 2 (GLS2) transcripts through RNA editing, reducing GLS2 expression and limiting lipid peroxidation, thereby enabling cells to evade ferroptosis. Restoration of ATXN8OS suppresses ADAR activity, stabilizes GLS2 expression, and promotes lipid reactive oxygen species (ROS) accumulation, ultimately resensitizing GBM cells to TMZ [49]. This mechanism illustrates how the loss of a regulatory lncRNA enhances ADAR-mediated RNA modification, thereby sustaining metabolic resistance and suppressing ferroptosis. Another mechanism of TMZ resistance involves the STAT3-mediated induction of the lncRNA LINC00520 and its interaction with the RBP Lin-28 Homolog B (LIN28B). Upon activation, STAT3 directly binds to the LINC00520 promoter, driving its overexpression in TMZ-resistant GBM cells. LINC00520, in turn, binds and stabilizes LIN28B, thereby inhibiting autophagy and reducing DNA damage, which collectively promote TMZ resistance. Silencing of LINC00520 resensitizes GBM cells to TMZ in vitro and in vivo, whereas its overexpression conferred resistance in vitro [50]. This mechanism highlights how a STAT3-driven lncRNA-RBP network can coordinately promote two key resistance processes: suppression of autophagy and attenuation of DNA damage.

### 7.2. Radioresistance

LncRNA-RBP interactions also contribute to radioresistance by reinforcing transcriptional programs that support the cellular response to DNA damage. DARS1 antisense RNA 1 (DARS-AS1) was identified through CRISPRi screening combined with multi-omics analyses as a critical lncRNA in GBM cells and GSCs. Depletion of DARS-AS1 impaired homologous recombination (HR)-mediated double-strand break (DSB) repair and enhanced radiosensitivity. Mechanistically, DARS-AS1 interacts with the RBP Y-box binding protein 1 (YBX1), enhancing its association with and stabilization of target mRNA, including key regulators of cell cycle progression, such as E2F1 and cyclin D1 (CCND1). In addition, the DARS-AS1/YBX1 complex stabilizes FOXM1 mRNA, a transcription factor implicated in GSC maintenance and DNA damage response [51]. This highlights that certain lncRNA-RPB interactions confer resistance to both therapy approaches and may therefore represent especially high-value therapeutic targets.

Extending beyond DNA repair mechanisms, lncRNAs can also drive radioresistance by modulating oncogenic signaling pathways. For example, LINC00839 influences the Wnt/β-catenin pathway and is upregulated in GSCs and recurrent GBM tissues. Its expression is regulated by METTL3-mediated m6A methylation, which promotes transcript stability through recruitment of the m6A reader YTHDF2. Functionally, LINC00839 serves as a scaffold that facilitates the interaction between β-catenin and the kinase c-Src, promoting β-catenin phosphorylation at Y654 and driving aberrant Wnt/β-catenin signaling. Notably, using Celecoxib, a Wnt/β-catenin inhibitor, can restore chemosensitivity and exhibit synergistic effects with radiation in GSC xenografts [52].

Collectively, the lncRNAs and RBPs described reveal a highly coordinated and multifaceted network of therapeutic resistance in GBM. These regulatory axes converge on shared oncogenic programs, including Wnt/β-catenin signaling, ERK/MAPK activation, DNA damage response, and suppression of ferroptosis, underscoring the interconnected nature of resistance mechanisms in this disease.

## 8. Immune Evasion

GBM is an immunologically “cold” tumor, characterized by a low tumor mutational burden and limited T cell infiltration [105,106]. Patients with GBM commonly exhibit systemic T cell lymphopenia, while tumor-infiltrating T cells display a profoundly exhausted phenotype [107,108]. Immunosuppression in GBM is driven by structural barriers within the tumor microenvironment and by the active production of immunosuppressive factors [109,110]. At the same time, immunosuppressive cell populations, including regulatory T cells (Tregs) and myeloid-derived suppressor cells (MDSCs), accumulate within the tumor microenvironment, further limiting effective antitumor immunity [111,112]. GBM also recruits macrophages and promotes their polarization toward an anti-inflammatory, tumor-supportive phenotype [113]. This profoundly immunosuppressive microenvironment has substantially limited the efficacy of immunotherapies in GBM, despite their success in other solid tumors such as melanoma and lung cancer [114,115,116,117,118]. Overcoming these therapeutic barriers will require a deeper understanding of the molecular mechanisms that drive immune evasion and suppression in GBM. In this context, interactions between RBPs and lncRNAs are emerging as important regulators of antitumor immunity and promising therapeutic targets for immunomodulatory strategies.

**Figure 4:**
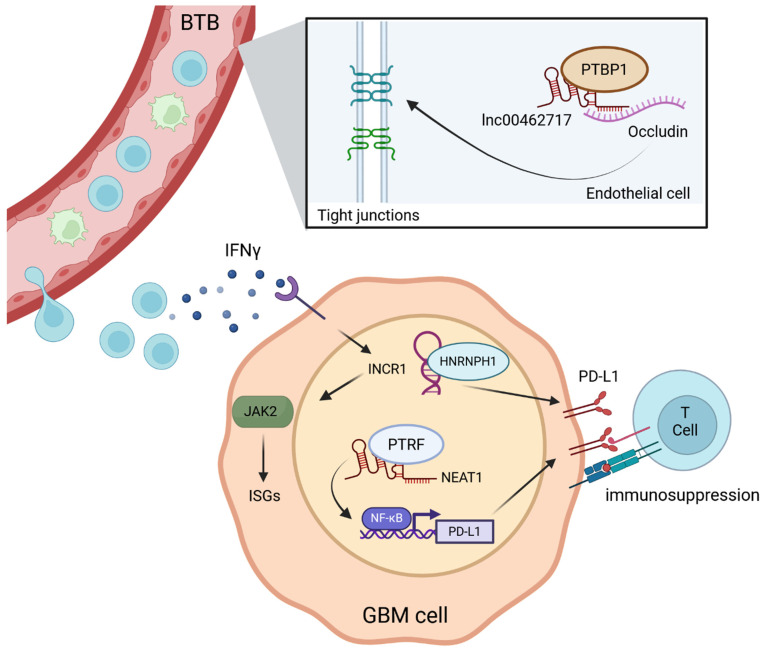
lncRNA-RBP networks mediating immune evasion in GBM. Lnc00462717 facilitates PTBP1 binding to Occludin mRNA, thereby enhancing Occludin stability and expression. INCR1 binds HNRNPH1, relieving HNRNPH1′s inhibitory effects on PD-L1 and JAK2 expression. PTRF stabilizes NEAT1, which suppresses UBXN1, thereby activating NF-κB signaling and promoting PD-L1 transcription. Created in BioRender. Mineo, M. (2026) https://BioRender.com/9vuvkx5.

Growing evidence indicates that interactions between RBPs and lncRNAs are crucial for maintaining the integrity of the BTB, which poses a significant hurdle to T cell infiltration and the success of immunotherapy. Specifically, Musashi RNA-binding protein 2 (MSI2) binds and stabilizes the lncRNA LINC00667, thereby enhancing the expression of tight junction proteins and decreasing BTB permeability [119]. Mechanistically, LINC00667 promotes degradation of IRF6 mRNA via the Staufen1-mediated mRNA decay pathway. When MSI2 is silenced, LINC00667 levels decrease, leading to IRF6 accumulation and increased BTB permeability [119]. Similarly, IGF2BP2, which is overexpressed in glioma endothelial cells, promotes tight junction protein expression and BTB formation by stabilizing the lncRNA FBXL19 antisense RNA 1 (FBXL19-AS1). This lncRNA binds to ZNF765 mRNA and decreases its expression through STAU1-mediated mRNA decay. Interestingly, ZNF765 acts as an inhibitor of IGF2BP2, creating a feedback loop that modulates BTB permeability [53]. Another lncRNA, lnc00462717, overexpressed in glioma endothelial cells, influences BTB permeability by facilitating PTBP1′s interaction with the 3′UTR of Occludin mRNA (Figure 4), a key tight junction protein. PTBP1 binding obstructs miR-186-5p-mediated downregulation of Occludin, contributing to junction integrity [54].

In addition to structural barriers, GBM reprograms the tumor immune microenvironment to favor immune evasion by expressing multiple immunosuppressive molecules, including the CD274 molecule (PD-L1) and CD47 [120,121]. PD-L1 functions as a key immune checkpoint ligand by engaging programmed cell death 1 (PD-1) on T cells, thereby suppressing their activation and effector functions [122]. CD47, in contrast, acts as a “don’t eat me” signal that inhibits phagocytosis by macrophages and other innate immune cells [123]. Emerging evidence indicates that several of these immune regulatory pathways are controlled by interactions between RBPs and lncRNAs. One example is the interferon-stimulated noncoding RNA 1 (INCR1), which is transcribed from the PD-L1 locus in response to IFNγ signaling. INCR1 enhances the expression of interferon-responsive and immunosuppressive genes by binding the RBP HNRNPH1 and relieving its inhibitory effects on the neighboring transcripts, PD-L1 and JAK2 (Figure 4). Because JAK2 is a central mediator of IFNγ/JAK-STAT signaling, INCR1 indirectly amplifies downstream immune checkpoint and immunosuppressive programs [55]. Importantly, INCR1 expression is increased in patients treated with IL-12 immune gene therapy, and its silencing sensitizes tumor cells to T cell-mediated cytotoxicity [124]. Therefore, INCR1 represents a promising target for enhancing the efficacy of immunotherapeutic approaches, including CAR T cell therapy and IL-12-based treatment strategies.

PD-L1 expression is also regulated transcriptionally through interactions between the lncRNA NEAT1 and Polymerase I and Transcript Release Factor (PTRF/Cavin-1) (Figure 4). PTRF functions as an RBP that stabilizes NEAT1, leading to suppression of the NF-κB inhibitor UBXN1. The resulting activation of NF-κB signaling promotes PD-L1 transcription and contributes to immune escape in GBM [56]. Notably, NF-κB is also a direct transcriptional activator of CD47, thereby contributing to tumor immune escape [125]. NEAT1 also plays a critical role in modulating the phenotype of tumor-associated macrophages (TAMs), which constitute up to 30–40% of the tumor mass and predominantly exhibit an anti-inflammatory, immunosuppressive phenotype [126,127]. Within the tumor microenvironment, NEAT1 is more highly expressed in TAMs than in cancer cells, and its levels correlate with patient response to immune checkpoint inhibitors [128].

HOTAIRM1, which was described as a pro-angiogenic lncRNA through its upregulation of IGFBP2 [33], also contributes to tumor-associated immunosuppression. Increased IGFBP2 expression contributes to the development of an immunosuppressive tumor microenvironment by driving macrophage polarization toward an anti-inflammatory phenotype [129]. Furthermore, inhibition of IGFBP2 in GBM cells enhances the expansion of both CD8+ and CD4+ T cells in vitro and in vivo, highlighting its role in suppressing anti-tumor immune responses [130]. Together, these findings highlight how a single interaction between a lncRNA and an RBP orchestrates multiple immunosuppressive signals, making them promising targets for immunotherapy.

## 9. Therapeutic Targeting of lncRNA-RBPs

**Figure 5:**
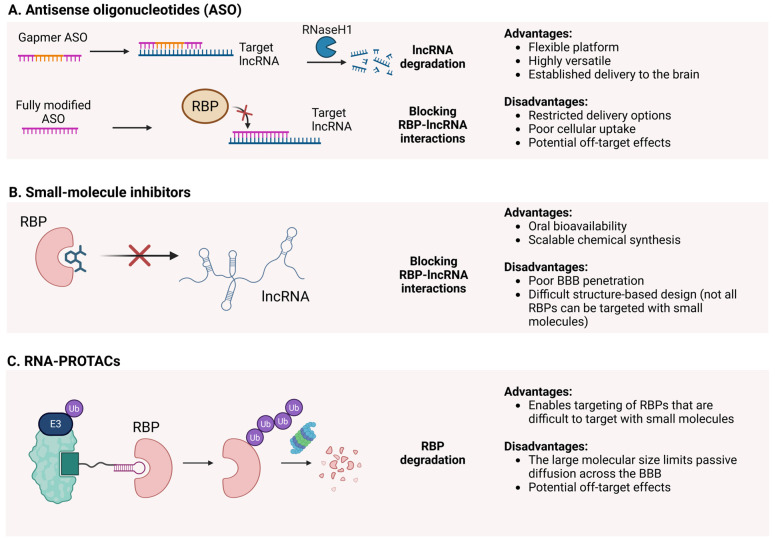
Therapeutic approaches for targeting lncRNA–RBP interactions. (**A**) Antisense oligonucleotides (ASOs) are chemically modified to enhance nuclease resistance and improve target affinity. Gapmer ASOs contain a central unmodified DNA sequence that recruits RNaseH1 and induces degradation of the target lncRNA. Fully modified ASOs bind the target lncRNA, thereby blocking its interaction with the RBP. (**B**) Small-molecule inhibitors bind the RNA-binding domains of RBPs to disrupt lncRNA-RBP interactions. (**C**) RNA-PROTACs are composed of an oligonucleotide that mimics the RNA motif recognized by the target RBP and a ligand that recruits an E3 ubiquitin (Ub) ligase, thereby inducing ubiquitination and subsequent proteasomal degradation of the target RBP. Created in BioRender. Mineo, M. (2026) https://BioRender.com/b9wj8js.

LncRNA-RBP networks have been implicated in virtually all GBM hallmarks, underscoring their potential as therapeutic targets. Emerging strategies to disrupt these oncogenic interactions include antisense oligonucleotides (ASOs) that silence pathogenic lncRNAs, small-molecule inhibitors that block RBP function or lncRNA-RBP binding, and proteolysis-targeting chimeras (PROTACs) that induce selective RBP degradation (Figure 5). Together, these strategies offer complementary approaches to target distinct components of the lncRNA-RBP regulatory axis in GBM.

### 9.1. Antisense Oligonucleotides

ASOs are short, single-stranded synthetic nucleic acids, typically 15–20 nucleotides long, that recognize complementary RNA sequences via Watson–Crick base pairing [131]. Because unmodified ASOs are highly susceptible to nuclease degradation and show poor cellular uptake, a range of chemical modifications has been developed to improve their pharmacokinetic and pharmacodynamic properties [132,133]. First-generation phosphorothioate (PS) ASOs, in which a non-bridging oxygen atom of the phosphodiester backbone is replaced with sulfur, exhibit enhanced nuclease resistance and prolonged systemic exposure [134,135]. Second-generation modifications, including 2′-O-methyl (2′-O-Me) and 2′-O-methoxyethyl (2′-MOE), improve target affinity while reducing nonspecific toxicity [136,137]. Third-generation chemistries, most notably locked nucleic acids (LNAs), further increase binding affinity and stability by conformationally constraining the ribose ring through a bridge linking the 2′-oxygen and 4′-carbon atoms [138].

The therapeutic activity of ASOs is largely determined by their structural architecture. Gapmer ASOs contain a central DNA region flanked by chemically modified nucleotides, commonly LNAs, which confer nuclease resistance and high-affinity target recognition. Upon hybridization to the target RNA, the DNA–RNA heteroduplex recruits RNase H1, leading to selective degradation of the transcript [139]. This mechanism is particularly attractive for eliminating oncogenic lncRNAs that function as molecular scaffolds within GBM-associated regulatory networks. In contrast, fully modified ASOs lack a central DNA gap and therefore do not activate RNase H. Instead, they act via steric blockade, preventing RBPs from interacting with their RNA targets without altering transcript abundance [140].

The clinical success of the fully modified 2′-MOE ASO nusinersen (Spinraza) highlights the therapeutic potential of steric-blocking ASOs. Nusinersen promotes exon 7 inclusion in SMN2 pre-mRNA by preventing the binding of the splicing repressors HNRNP A1/A2, thereby restoring functional SMN protein expression in spinal muscular atrophy [141,142]. A similar strategy could be applied to oncogenic lncRNA-RBP complexes in GBM, in which ASOs are designed to occupy critical RBP-binding motifs and disrupt pathogenic interactions without degrading the lncRNA. This concept has been demonstrated for INCR1, where a fully modified ASO targeting the HNRNPH1-binding region disrupted RBP recruitment and attenuated downstream immune-evasion signaling [55]. Collectively, both gapmer and steric-blocking ASOs represent promising approaches for therapeutically targeting oncogenic lncRNA-RBP networks in GBM.

### 9.2. Small-Molecule Inhibitors

Small molecules offer several advantages over ASOs, including oral bioavailability and scalable chemical synthesis [143]. However, achieving sufficient BBB penetration remains a major obstacle for their application in GBM. Mechanistically, small molecules can disrupt lncRNA-RBP interactions either by directly inhibiting RBPs through their RNA-binding domains (RBDs) or by targeting lncRNA structural motifs that function as RBP docking platforms [144,145].

Direct inhibition of RBDs has been demonstrated for several members of the heterogeneous nuclear ribonucleoprotein (hnRNP) family. Using structure-guided drug design, Carabet et al. identified VPC-80051, the first reported small-molecule inhibitor of the HNRNPA1 RBD. VPC-80051 binds the UP1 domain of HNRNPA1 and suppresses production of the oncogenic AR-V7 splice variant in castration-resistant prostate cancer [146]. Similarly, the compounds 2155-14 and 2155-18 were shown to bind HNRNPH1, reduce its expression, and induce apoptosis in melanoma cells [147,148]. Together, these studies provide proof-of-principle that RBPs, historically considered challenging therapeutic targets, can be pharmacologically modulated by small molecules. Future studies should determine whether these compounds, or next-generation derivatives, can selectively disrupt oncogenic lncRNA-RBP interactions that contribute to GBM pathogenesis.

An alternative strategy focuses on targeting lncRNA structure rather than the protein partner. RNA G-quadruplexes (rG4s), four-stranded guanine-rich secondary structures, are recognized by numerous RBPs and frequently function as high-affinity protein-binding platforms [149]. Small molecules that bind rG4s within metastasis-associated lung adenocarcinoma transcript 1 (MALAT1) have been shown to disrupt the MALAT1-non-POU domain-containing octamer binding (NONO) interaction, demonstrating the feasibility of selectively perturbing lncRNA-RBP networks through RNA structure targeting [150]. Notably, several lncRNAs discussed in this review, including NEAT1 and HOTAIR, contain rG4 motifs, raising the possibility that G-quadruplex-targeting ligands could be leveraged to interfere with their RBP interactions [149].

A further extension of this concept is the development of Ribonuclease-Targeting Chimeras (RIBOTACs), bifunctional small molecules that simultaneously bind a specific RNA structure and recruit endogenous RNase L, thereby inducing catalytic RNA degradation [151]. A recently reported RIBOTAC targeting rG4 motifs within the lncRNA TERRA achieved selective TERRA degradation and disrupted telomere maintenance [152]. Applied to GBM-associated lncRNAs, RIBOTAC-based approaches could not only disrupt individual lncRNA-RBP interactions but also eliminate the lncRNA scaffold itself, thereby dismantling broader oncogenic lncRNA-RBP regulatory networks.

### 9.3. Proteolysis-Targeting Chimeras

PROTACs are heterobifunctional molecules composed of two ligands connected by a chemical linker: one engages a protein of interest, while the other recruits an E3 ubiquitin ligase, thereby inducing ubiquitination and subsequent proteasomal degradation of the target protein [153]. As noted above, although certain RBPs, including HNRNPA1 and HNRNPH1, have been pharmacologically engaged by small molecules that could in principle be adapted into PROTACs, the broader application of this strategy remains limited by the scarcity of high-affinity, selective ligands for most RBPs.

To address this limitation, RNA-PROTACs have been developed by replacing the conventional small-molecule protein-binding moiety with an oligonucleotide that mimics the RNA element recognized by the target RBP. In a seminal study, Ghidini et al. demonstrated this concept by conjugating the Lin28A-binding RNA sequence to a von Hippel–Lindau (VHL) E3 ligase–recruiting peptide, thereby generating an RNA-PROTAC that selectively binds LIN28A and promotes its ubiquitin-dependent proteasomal degradation in cancer cell models [154]. This strategy has since been extended to additional nucleic acid-binding proteins, further supporting the generalizability of oligonucleotide-based PROTAC approaches [155,156,157].

Despite these advances, the application of RNA-PROTACs in GBM remains largely unexplored, even though this disease is characterized by widespread dysregulation of RBPs, including LIN28B, which has been implicated in temozolomide resistance [50,158]. This translational gap likely reflects a broader pharmacological challenge, as the relatively large molecular size of PROTACs and related constructs limits passive diffusion across the BBB. Emerging delivery strategies, including nanoparticle-based systems, receptor-mediated transcytosis, and convection-enhanced delivery, may help overcome this limitation [159]. Integrating such approaches with RNA-PROTAC platforms could therefore represent a promising direction for targeting dysregulated lncRNA-RBP networks in GBM.

Taken together, ASOs, small-molecule inhibitors, and PROTACs represent three mechanistically distinct strategies for targeting lncRNA-RBP networks that underpin GBM hallmarks. Although challenges related to BBB penetration, tumor heterogeneity, and off-target effects remain, advances in RNA therapeutics and targeted protein degradation are rapidly improving the feasibility of translating lncRNA-RBP-directed therapies into clinical applications for GBM. Emerging delivery approaches, such as lipid nanoparticles and convection-enhanced delivery, may improve target engagement within the central nervous system but require further optimization for lncRNA-RBP-targeted interventions. Achieving tumor specificity remains a critical hurdle, as oncogenic lncRNAs such as NEAT1, MALAT1, and HOTAIR also perform important physiological functions in normal tissues. Moreover, the extensive interconnectedness of lncRNA-RBP networks, in which individual RBPs interact with multiple lncRNAs and vice versa, suggests that combinatorial therapeutic approaches may be necessary to achieve durable suppression of oncogenic signaling. Continued advances in target identification, delivery technologies, and network-level therapeutic design are likely to position lncRNA-RBP-directed strategies as an important component of future precision medicine approaches for GBM.

## 10. Conclusions and Future Directions

GBM progression is driven by a complex network of molecular interactions that promote cell proliferation, angiogenesis, invasion, resistance to therapy, and immunosuppression. Growing evidence suggests lncRNA-RBP interactions as key regulatory nodes underlying these GBM hallmarks. By modulating multiple signaling pathways, lncRNA-RBP complexes sustain oncogenic programs that drive tumor progression. Notably, many of these interactions influence multiple cancer hallmarks simultaneously, underscoring extensive crosstalk among pathways. Given their central role in GBM pathophysiology, lncRNA-RBP interactions represent attractive therapeutic targets. Emerging approaches, including small-molecule inhibitors, antisense oligonucleotides, and RNA-PROTACs, have demonstrated encouraging potential. However, translating these strategies to brain tumors remains challenging due to obstacles related to delivery across the BBB, target specificity, and therapeutic efficacy. Furthermore, despite significant progress in elucidating individual lncRNA-RBP regulatory axes, the broader architecture of these networks remains incompletely defined. In particular, the contributions of context-dependent interactions, intratumoral heterogeneity, and dynamic network rewiring during disease progression and treatment response require further investigation. Future studies integrating high-throughput interactome mapping, spatial multi-omics, and rigorous functional validation will be critical for delineating the full spectrum of lncRNA-RBP regulatory networks in GBM. A more comprehensive understanding of these interactions will not only provide deeper insight into GBM biology but may also facilitate the discovery of novel therapeutic vulnerabilities for this highly aggressive and currently incurable malignancy.

## Figures and Tables

**Table 1:** Overview of lncRNA-RBP interaction networks regulating the hallmarks of GBM.

RBP	LncRNA	Hallmark	Reference
DHX9	HIF1A-AS2	Sustained proliferation	[21]
IGF2BP2	HIF1A-AS2	Sustained proliferation	[21]
ACTN4	SChLAP1	Sustained proliferation, Invasion	[27]
HNRNPL	SChLAP1	Sustained proliferation, Invasion	[27]
PTBP1	LINREP	Sustained proliferation, Invasion	[28]
UPF1	LINREP	Sustained proliferation, Invasion	[28]
CBX3	LINC00998	Sustained proliferation	[29]
SRSF1	NEAT1	Sustained proliferation	[30]
EIF4A3	LINC00680	Sustained proliferation, Invasion	[31]
	TTN-AS1	Sustained proliferation, Invasion	[31]
UPF1	LINC00313	Sustained proliferation, Invasion	[32]
METTL3	HOTAIRM1	Angiogenesis, Immune evasion	[33]
IGF2BP2	OIP5-AS1	Angiogenesis, Therapeutic resistance	[34,35]
ZRANB2	SNHG20	Angiogenesis, Invasion	[36]
PABPC5	HCG15	Angiogenesis	[37]
TIAR	LOXL1-AS1	Angiogenesis, Invasion	[38]
HNRNPD	LINC00707	Angiogenesis	[39]
CBP/p300	LUCAT1	Metabolic alteration	[40]
LIN28A	SNHG14	Metabolic alteration	[41]
IGF2BP2	CASC9	Metabolic alteration	[42]
FUS	LINC00470	Metabolic alteration	[43]
CBP	LINC01057	Phenotypic plasticity	[44]
YWHAE	MIR222HG	Phenotypic plasticity	[45]
PTBP1	MIR155HG	Therapeutic resistance	[46]
IGF2BP3	RMRP	Therapeutic resistance	[47]
IGF2BP2	DANCR	Therapeutic resistance	[48]
ADAR	ATXN8OS	Therapeutic resistance	[49]
LIN28B	LINC00520	Therapeutic resistance	[50]
YBX1	DARS-AS1	Therapeutic resistance	[51]
YTHDF2	LINC00839	Therapeutic resistance	[52]
IGF2BP2	FBXL19-AS1	Immune evasion	[53]
PTBP1	lnc00462717	Immune evasion	[54]
HNRNPH1	INCR1	Immune evasion	[55]
PTRF	NEAT1	Immune evasion	[56]

## Data Availability

No new data were created or analyzed in this study. Data sharing is not applicable to this article.

## Abbreviations

The following abbreviations are used in this manuscript:

2′-O-Me	2′-O-methyl
2′-MOE	2′-O-methoxyethyl
AC-like	astrocyte-like
ACTN4	actinin alpha 4
ADAR	adenosine deaminase acting on RNA
ASO	antisense oligonucleotide
ATXN8OS	ataxin-8 opposite strand
BBB	blood–brain barrier
BTB	blood-tumor barrier
CASC9	cancer susceptibility candidate 9
CBP	CREB-binding protein
CBX3	chromobox 3
CCND1	cyclin D1
CDK	cyclin-dependent kinase
ceRNA	competing endogenous RNA
DANCR	differentiation antagonizing non-protein coding RNA
DARS-AS1	DARS1 antisense RNA 1
DHX9	ATP-dependent RNA helicase A
DSB	double-strand break
ECM	extracellular matrix
EGR3	early growth response 3
EIF4A3	eukaryotic translation initiation factor 4A3
FBXL19-AS1	FBXL19 antisense RNA 1
FGFR1	fibroblast growth factor receptor 1
FOXK1	forkhead box K1
GBM	glioblastoma
GLUT	glucose transporters
GLS2	glutaminase 2
GSC	glioma stem cells
HCG15	human leukocyte antigen complex group 15
HDAC	histone deacetylase
HIF-1α	hypoxia-inducible factor-1α
HIF1A-AS2	hypoxia-inducible factor 1 alpha-antisense RNA 2
HK	hexokinase
HMGA1	high mobility group AT-hook 1
hnRNP	heterogeneous nuclear ribonucleoprotein
HOTAIRM1	HOXA transcript antisense RNA, myeloid-specific 1
HR	homologous recombination
HuR	human antigen R
IGF2BP2	insulin-like growth factor 2 mRNA-binding protein 2
IGFBP2	insulin-like growth factor binding protein 2
INCR1	interferon-stimulated noncoding RNA 1
IRF6	interferon regulatory factor 6
Lin28A	lin-28 RNA-binding posttranscriptional regulator A
LIN28B	lin-28 homolog B
LINC00707	long intergenic non-coding RNA 707
LNA	locked nucleic acid
lncRNAs	long non-coding RNAs
LOXL1-AS1	LOXL1 antisense RNA 1
LUCAT1	lung cancer-associated transcript 1
MALAT1	metastasis-associated lung adenocarcinoma transcript 1
MDSCs	myeloid-derived suppressor cells
MES-like	mesenchymal-like
METTL3	methyltransferase-like 3
MIR155HG	MIR155 host gene
MIR210HG	MIR210 host gene
MIR222HG	miR222/221 cluster host gene
MMP	matrix metalloproteinase
MSI2	Musashi RNA-binding protein 2
NEAT1	nuclear paraspeckle assembly transcript 1
NONO	non-POU domain containing octamer binding
NPC-like	neural-progenitor-like
OCT1	POU class 2 homeobox 1
OIP5-AS1	OIP5 antisense RNA 1
OPC-like	oligodendrocyte-progenitor-like
OS	overall survival
PABPC5	poly(A)-binding protein cytoplasmic 5
PD-1	programmed cell death 1
PD-L1	CD274 molecule
PFS	progression-free survival
PID1	phosphotyrosine interaction domain containing 1
PKM2	pyruvate kinase M2
PKP2	plakophilin 2
PMT	proneural-to-mesenchymal transition
Pol II	RNA polymerase II
PROTAC	proteolysis-targeting chimeras
PS	phosphorothioate
PTBP1	polypyrimidine tract-binding protein 1
PTRF	polymerase I and transcript release factor
RBD	RNA-binding domains
RBPs	RNA-binding proteins
rG4	RNA G-quadruplex
RIBOTAC	ribonuclease-targeting chimera
RMRP	RNA component of the mitochondrial RNA processing endoribonuclease
ROS	reactive oxygen species
RTN4	reticulon 4
SChLAP1	SWI/SNF complex antagonist associated with prostate cancer 1
SHCBP1	SHC binding and spindle-associated 1
SNHG14	small nucleolar RNA host gene 14
SNHG20	small nucleolar RNA host gene 20
SPI1	spi-1 proto-oncogene
SRSF1	serine and arginine-rich splicing factor 1
STAU1	Staufen double-stranded RNA binding protein 1
SUMO	small ubiquitin-like modifier
TAM	tumor-associated macrophages
TCA	tricarboxylic acid
TIAR	TIA1-related protein
TME	tumor microenvironment
TMZ	temozolomide
Tregs	regulatory T cells
TTF	tumor-treating fields
TTN-AS1	TTN antisense RNA 1
UPF1	UPF1 RNA helicase and ATPase
VHL	von Hippel–Lindau
VM	vasculogenic mimicry
YBX1	Y-box binding protein 1
YWHAE	tyrosine 3-monooxygenase/tryptophan 5-monooxygenase activation protein epsilon
ZHX2	zinc fingers and homeoboxes 2
Zic4	zinc family zinc finger 4
ZNF331	zinc-finger protein 331
ZNRF3	zinc and ring finger 3
ZRANB2	zinc finger RANBP2-type containing 2
